# RACIAL AND ETHNIC DISPARITIES IN NEIGHBORHOOD SOCIOECONOMIC CHARACTERISTICS ASSOCIATIONS WITH VISCERAL FAT

**DOI:** 10.1093/geroni/igad104.0996

**Published:** 2023-12-21

**Authors:** Shawna Follis, Jeremey Shropshire

**Affiliations:** Stanford University, Stanford, California, United States; Morehouse University, Atlanta, Georgia, United States

## Abstract

Visceral adipose tissue (VAT) has a more significant role in the pathophysiology of cardiometabolic diseases than subcutaneous adipose tissue (SAT). Our previous findings suggest VAT represents an important pathway through which racial disparities in the social environment are biologically embodied. To understand the role of structural racism in this pathway, this paper investigated racial disparities in neighborhood socioeconomic status (NSES) and VAT. We hypothesized that NSES is inversely associated with VAT and VAT/SAT ratio over 6 years and associations differ by race and ethnicity. Abdominal VAT and SAT area (cm2) were measured at years 0, 3, and 6 using dual-energy X-ray absorptiometry in a subsample (n=11,020) of the Women’s Health Initiative (age 50-79). The NSES Z-scores were obtained from the US Census and American Community Survey based on participant’s census tract residence. We evaluate racial and ethnic disparities in the exposure and outcome using ANOVA, and associations between NSES with VAT using mixed effects models. We found that NSES and VAT area differed by race and ethnicity. American Indian and Alaska Native (AIAN), Black, and Latina groups were significantly (P< 0.01) more likely to live in neighborhoods with NSES Z-scores below the population mean (Z-score=0) compared with White and Asian races. AIAN, Latina, and Black women had the highest VAT (220 cm2, 185 cm2, 177 cm2, respectively). Findings support the hypothesis that associations between NSES and VAT differ by race and ethnicity. Results from mixed effects models are expected to provide longitudinal evidence that structural determinants are associated with VAT.

